# Twist promotes tumor metastasis in basal-like breast cancer by transcriptionally upregulating ROR1: Erratum

**DOI:** 10.7150/thno.56462

**Published:** 2021-01-01

**Authors:** Jingying Cao, Xin Wang, Tao Dai, Yuanzhong Wu, Meifang Zhang, Renxian Cao, Ruhua Zhang, Gang Wang, Rou Jiang, Binhua P. Zhou, Jian Shi, Tiebang Kang

**Affiliations:** 1 State Key Laboratory of Oncology in South China, Collaborative Innovation Center for Cancer Medicine, Sun Yat-Sen University Cancer Center, Guangzhou 510060, P. R. China.; 2 Markey Cancer Center, The University of Kentucky, College of Medicine, Lexington, Kentucky 40506, USA.; 3 Department of Medicine Clinical Laboratory, The Third Xiangya Hospital of Central South University, Changsha, 410013, P. R. China.; 4 Department of Urology, The Affiliated Cancer Hospital of Xiangya School of Medicine of Central South University, Hunan Cancer Hospital, Changsha, 410013, P. R. China.; 5 Institute of Clinical Medicine, the First Affiliated Hospital of University of South China, Hengyang, P. R. China.; 6 Department of Pathology, School of Basic Medical Science; Guangdong Province Key Laboratory of Molecular Tumor Pathology, Southern Medical University, Guangzhou 510515, China

We noticed an error in Figure 4G, where the HE (200 x) for shROR1+Twist was misplaced with the same HE picture for Twist. The figure with the error corrected is shown below. The correction made in this erratum does not affect the original conclusions or any part of the text and figure legends. The authors wish to apologize for any inconvenience or misunderstanding that this error may have caused.

## Figures and Tables

**Figure 4:**
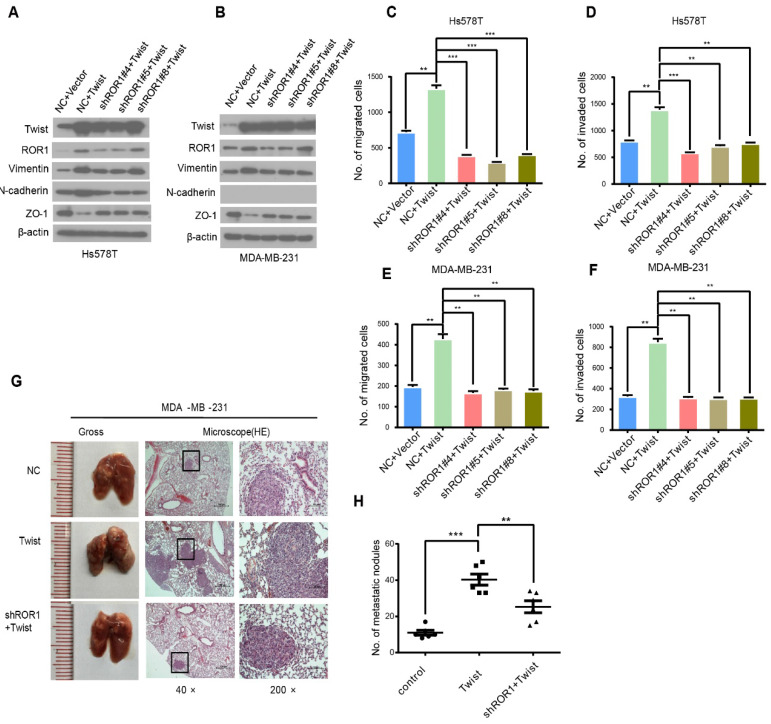
** The promotion of cell migration, invasion and cancer metastasis after Twist overexpression primarily depends on ROR1. (A-B)** The indicated molecules were analyzed by Western blot in the Hs578T and MDA-MB-231 cells stably expressing control, ROR1 shRNA, Twist, or both, as indicated. **(C-F)** Cell migration and invasion were determined in the indicated stable cell lines. The results are expressed as the mean ± SD of three independent experiments. * P < 0.05, ** P < 0.01 and *** P < 0.001 using Student's *t-*test. **(G-H)** An in vivo lung metastasis model was established in nude mice using MDA-MB-231 cells stably expressing control, ROR1 shRNA, Twist, or both, as indicated. **(G)** Representative results of gross and H&E staining (middle scale: 40×; right scale: 200×) of metastatic lung nodules. Scale bars, 40×,500μm; 200×,100μm. **(H)** Illustration of the statistical results (n = 6). The results are expressed as the mean ± SD. * P < 0.05, *** P < 0.001 using Student's *t-*test.

